# Mental Stress Assessment and Clinical Application of Wearable Devices as Evaluable Outcomes in Robotic Prostatectomy

**DOI:** 10.1245/s10434-025-18914-1

**Published:** 2025-12-19

**Authors:** Taku Naiki, Yoshihisa Mimura, Yosuke Sugiyama, Toshiki Etani, Akihiro Nakane, Takashi Nagai, Yoshihiko Tasaki, Nobuhikio Shimizu, Masakazu Gonda, Maria Aoki, Toshiharu Morikawa, Shoichiro Iwatsuki, Shuzo Hamamoto, Yukihiro Umemoto, Takahiro Yasui

**Affiliations:** 1 Department of Nephro-urology, Nagoya City University, Graduate School of Medical Sciences, Nagoya, Japan; 2 Department of Urology, Nagoya City University West Medical Center, Nagoya, Japan; 3 Department of Pharmacy, Nagoya City University Hospital, Nagoya, Japan; 4 Department of Urology, Gamagori City Hospital, Gamagori City, Japan

**Keywords:** Education program, Heart rate variation, Mental stress, Robot-assisted radical prostatectomy, Wearable device

## Abstract

**Background:**

Because of the rapid uptake of robot-assisted radical prostatectomy (RARP), educational programs or established methods based on the skill level and mental stress of surgeons have yet to be established. This study aimed to measure physiologic stress as heart rate (HR) changes and heart rate variations (HRVs) in surgeons wearing a device during RARP.

**Patients and Methods:**

We collected device data for 30 consecutive cases from surgeon A, relatively inexperienced in RARP, and surgeon B, experienced in over 200 cases. As a wearable device, we used Fitbit Charge 2 (Fitbit Inc., San Francisco, CA, USA). Surgical outcomes included estimated blood loss volume and robotic console time; HR changes and HRVs in each surgeon were measured. The standard deviation of NN intervals (SDNN) for HRV was calculated and cumulative sum (CUSUM) control charts used to quantitatively evaluate surgeons’ learning curves.

**Results:**

For surgeon A, as case numbers increased, console time was significantly reduced; maximum and average HRs were also significantly decreased. However, a trend was not observed for surgeon B. The SDNN, as a biomarker of mental stress in surgeon B, was significantly better compared with surgeon A. For surgeon A, according to an analysis using CUSUM methods, and average and maximum HRs, learning curves with regard to console time and estimated blood loss volume were similar.

**Conclusions:**

By using a wearable device, mental stress, as represented by the HRV, could be easily estimated and visualized as a surgical outcome. This affected surgeons’ learning curves, including for console time and estimated blood loss volumes.

Robot-assisted radical prostatectomy (RARP) is considered a feasible and safe procedure that can lead to clinical and oncological outcomes for patients with localized prostate cancer. Even for novice surgeons, previous reports described how the learning curve for RARP was shorter than for a straightforward laparoscopic procedure; therefore, RARP techniques have emerged and been adopted at an unprecedented pace in daily clinical practice. However, educational programs and methods established on the basis of the specific level of skill and mental stress experienced by each surgeon do not exist. For the establishment of an adequate educational program, the varied surgical outcomes that are normally obtained should be analyzed. However, almost all studies have considered different variables with regard to the quality of surgery according to effectiveness, or perioperative and postoperative outcomes on the patient’s side.^1,2^ Studies that focus on a surgeon’s well-being and comfort during surgery are rare.

Several articles have described how surgeons who must master new tasks to safely complete a procedure tend to experience acute mental stress.^3–5^ Growing evidence suggests that, in the operating room, surgeons experience excessive levels of stress, including mental stress, thought to be detrimental to their skills.^6–9^ In robotic surgery, physical stress tends to be reduced compared with laparoscopic surgery.^10^ However, a previous report described how a disruption in the flow of a surgical procedure can frequently occur and is associated with an increased mental workload even in robotic surgery.^11^ In addition, the precise and real-time evaluation of mental stress in a surgeon is very difficult.

Recently, interest has grown in using wrist-worn wearable devices, based on an optical pulse photoplethysmography (PPG) and three-dimensional (3D) accelerometer, to measure movement that yields biometric information in real time. To date, in using these wearable devices, several reports described the usefulness of evaluating patients receiving treatment, including surgery and chemotherapy.^12–17^ Furthermore, evidence is growing for the feasibility of measuring parameters such as heart rate (HR) with these devices to estimate the mental health status of patients with mental illness.^18^ However, to our knowledge, no study has taken into consideration the evaluation of the mental status of surgeons of RARP using such wearable devices.

Therefore, in this study, we tried to measure surgeons’ HRs, which reflect physiologic stress, during RARP using a wearable device and investigated whether such parameters could become evaluable for surgical outcomes in terms of learning curves.

## Patients and Methods

### Patient Enrollment

We enrolled 180 patients who underwent RARP at Nagoya City University and affiliated hospitals between June 2022 and April 2023. We prospectively collected and assessed data concerning patients’ basic characteristics, including age, body mass index (BMI), prostate volume, and surgical outcomes. To equalize the data as much as possible, focusing on low and intermediate risk patients, we collected device data for 30 consecutive cases from surgeon A, who had just started performing RARP, and surgeon B, who had performed RARP in over 200 cases. As a wearable device, a Fitbit Charge 2 (Fitbit Inc., San Francisco, CA, USA) was used. Surgical outcomes included estimated blood loss volume and robotic console time, and from HR changes in surgeons, the HR variation (HRV) was obtained. The ethics committees (approval no. 60-23-0115) gave approval for this study and opt-out information for patients was available on websites. The study was performed in accordance with the Declaration of Helsinki (2013).

### Heart Rate Monitoring and Variability

As a PPG sensor in this study, a Fitbit Charge 2, which is a wrist-worn wearable device, was used and the HR was measured. The Fitbit Charge 2 monitors HR activity using PPG technology to allow real-time HR recording. Each surgeon wore a Fitbit Charge 2 during surgery that recorded the average HR per minute every 5 min. For the analysis of HRVs, we used the method of Nakagome et al.^18^ Briefly, 60/mean HR obtained from the Fitbit Charge 2 was considered a surrogate for RR intervals recorded by an electrocardiogram (ECG). We evaluated the standard deviation of NN intervals (SDNN) for an initial 15, 30, and 45 min of surgery. The SDNN was calculated as the standard deviation of the RR interval. In addition, for the analysis of differences in each section in a RARP surgical procedure, and using process mapping of the RARP procedure as previously described,^19^ we divided each section as outlined below and examined the HRVs in surgeon B: section 1, set-up of operative field; section, exposure and dissection of anterior prostate; section, bladder neck transection; section, dissection of seminal vesicles and posterior prostate; section, finalize the dissection; and section, anastomosis.

### Evaluating Learning Curves Using a Cumulative Sum Control Charts Method for Evaluating Surgical Performance

For the evaluation of learning curves for surgeon A, a cumulative sum control charts (CUSUM) method was used to quantitatively evaluate the learning curves of surgeons.^20^ CUSUM is the running sum of the differences between individual data points and the mean of all data points. CUSUM at each time point was plotted to create learning curves. Robotic console time, blood loss volume, preoperative HR, and average and maximum HRs during surgery were used to construct learning curves. Each inflection point was defined as the number of cases required for the surgeon to master an RARP technique. To identify the inflection point of each learning curve, we performed joinpoint liner regression analysis using a Joinpoint Regression Program, Version 5.2.0.0 April, 2024 (Statistical Research and Applications Branch, National Cancer Institute).

### Statistics

Data were determined as means with 95% confidence intervals (CI), medians, ranges, or frequencies (%). Statistical significance was set as *p* < 0.05. Differences in the continuous variables were compared using Mann–Whitney *U* and Kruskal Wallis tests followed by Dunn’s test. The relationship between the two variables was assessed by calculating Pearson’s correlation coefficient and performing liner regression analysis. For an analysis of the impact of factors related to patients on surgeons, univariate logistic regression analyses of risk factors for poorer SDNN15 were analyzed. As variables that were clinically important factors, the median age of patients (71 years old), median BMI (24.2 kg/m^2^), median prostate volume of patients (44 mL), and surgeons were included. Statistical analyses were performed using the following software: GraphPad Prism 9 (GraphPad Software, Boston, MA, USA) and EZR (Saitama Medical Center, Jichi Medical University, Saitama, Japan).^21^ Designated data were assessed using EZR software.

## Results

### Patient’s Surgical Outcomes and HRV Data between Surgeons A and B

**Fig. 1:**
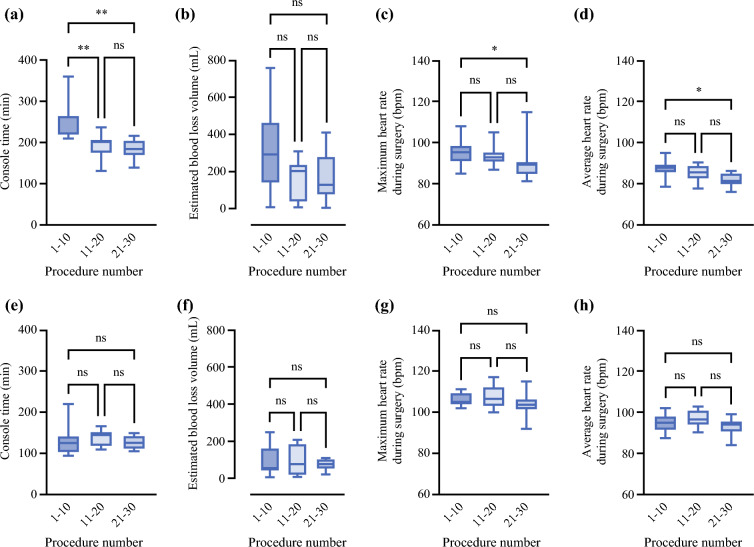
Comparison of various parameters for every ten surgeries between surgeons A and B; console time, estimated blood loss volume, maximum and average heart rates during surgery by surgeons A (**a**–**d**) and B (**e**–**h**) were evaluated; the horizontal midline in each box denote the median value; the bottom and top of each box represent the 25^th^ and 75^th^ percentiles, respectively; whisker ends designate minimum and maximum values of all data. *p < 0.05; **p < 0.01; *bpm* beats per minute, *ns* not significant

**Fig. 2:**
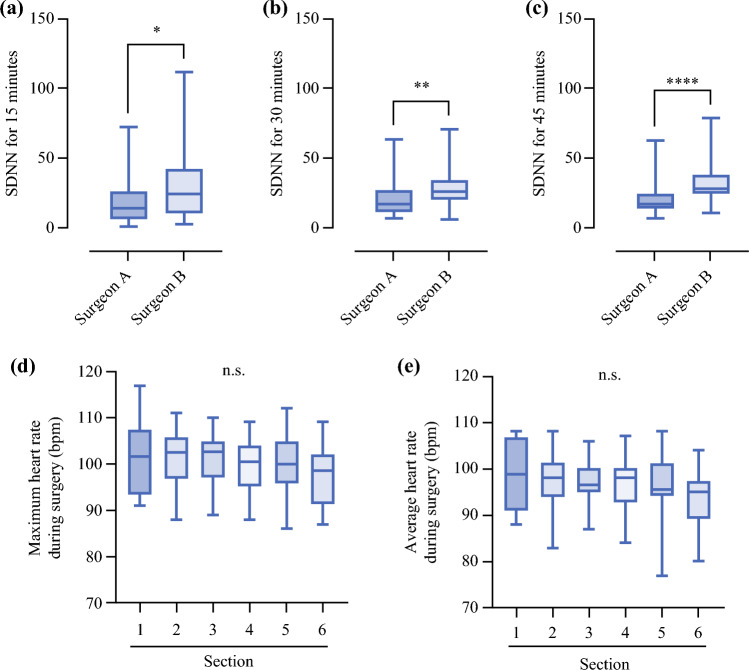
Comparison of heart rate viability between two surgeons and of heart rate for each surgical procedure by surgeon B; the SDNNs at 15 (**a**), 30 (**b**), and 45 minutes (**c**) from the start of surgery were calculated; the horizontal midline in each box denote the median value; the bottom and top of each box represent the 25^th^ and 75^th^ percentiles, respectively; whisker ends designate minimum and maximum values of all data; maximum heart rate (**d**) and average heart rate (**e**) in each section of RARP; the horizontal midline in each box denoted the median value; the bottom and top of each box indicate the 25^th^ and 75^th^ percentiles, respectively; the whisker ends indicate the minimum and maximum values of all data, respectively; *p < 0.05; **p < 0.01; ****p < 0.0001; *bpm* beats per minute, *ns* not significant, *RARP* robot-assisted radical prostatectomy, *SDNN* standard deviation of NN intervals

Table 1 shows a direct comparison between the two surgeons: median robotic console time and estimated blood loss volume were significantly better for surgeon B compared with surgeon A (*p* < 0.001). With regard to individual differences, the HRV, including the median average HR and median maximum HR, was significantly higher for surgeon B compared with surgeon A. For an analysis of the data of each surgeon for every ten cases, a tendency for a significant improvement with each subsequent case was observed in the median robotic console time for surgeon A (Fig. 1a). However, the estimated blood loss volume remained unchanged (Fig. 1b). In terms of HRVs, both median maximum and average HRs for surgeon A were significantly lower for 21–30 cases than for 1–10 cases (Fig. 1c,d). In comparison, for surgeon B, no trends were observed in median console time, estimated blood loss volume, and HRVs for every ten cases (Fig. 1e–h). When evaluating the SDNN, which is an index of mental stress, even though setting some baselines during calculations, every median SDNN for surgeon B was significantly better compared with those for surgeon A (Fig. 2a–c). In addition, for the evaluation of the HRV of each section of surgical procedures of RARP for surgeon B, both the median maximum and average HRs were unchanged in a total of 30 cases (Fig. 2d,e).

**Table 1:** Comparison of console time, estimated blood loss volume, preoperative heart rate, average and maximum heart rate during surgery, and patients’ characteristics between two group of surgeries in each surgeon

Characteristics	RARP performed by surgeon A (*n* = 30)	RARP performed by surgeon B (*n* = 30)	*p*-Value
Median age of patients, years (range)	70 (59–77)	72 (57–79)	0.59
Median BMI of patients, kg/m^2^ (range)	22.3 (18.6–29.6)	24.5 (17.2–28.7)	0.26
Median prostate volume of patients, mL (range)	44.0 (27.0–103.0)	44.0 (27.0–88.0)	0.91
Median console time, min (range)	202 (131–360)	130(94–221)	< 0.001
Median estimated blood loss volume, mL (range)	205 (0–758)	51 (2–250)	< 0.001
Median average HR, bpm (range)	85 (76–94)	94 (84–102)	< 0.001
Median maximum HR, bpm (range)	92 (81–115)	105 (92–117)	< 0.001
Median preoperative HR, bpm (range)	70 (66–77)	76 (67–83)	< 0.001

*BMI* body mass index, *bpm* beat per minute, *HR* heart rate

### Evaluation of HRV for Surgical Outcomes Using CUSUM Analysis for a Learning Curve

**Fig. 3:**
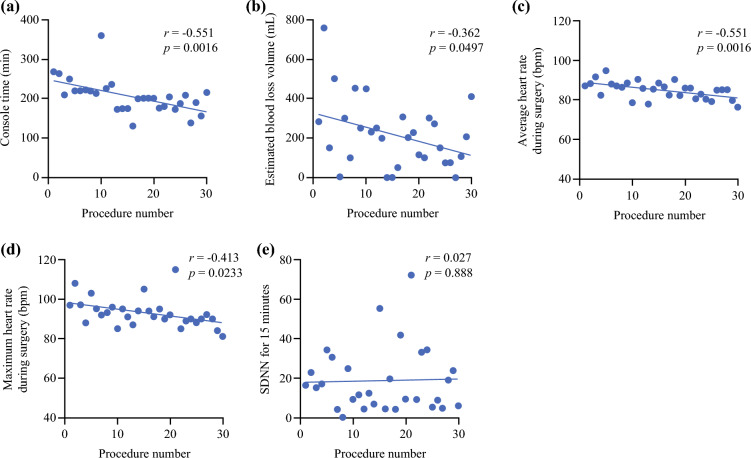
Pearson’s correlation coefficients (*r*) and *p*-values between procedure number and console time (**a**), estimated blood loss volume (**b**), average (**c**) and maximum heart rates during surgery (**d**), and standard deviation of the NN intervals (SDNN) for 15 min (**e**)

**Fig. 4:**
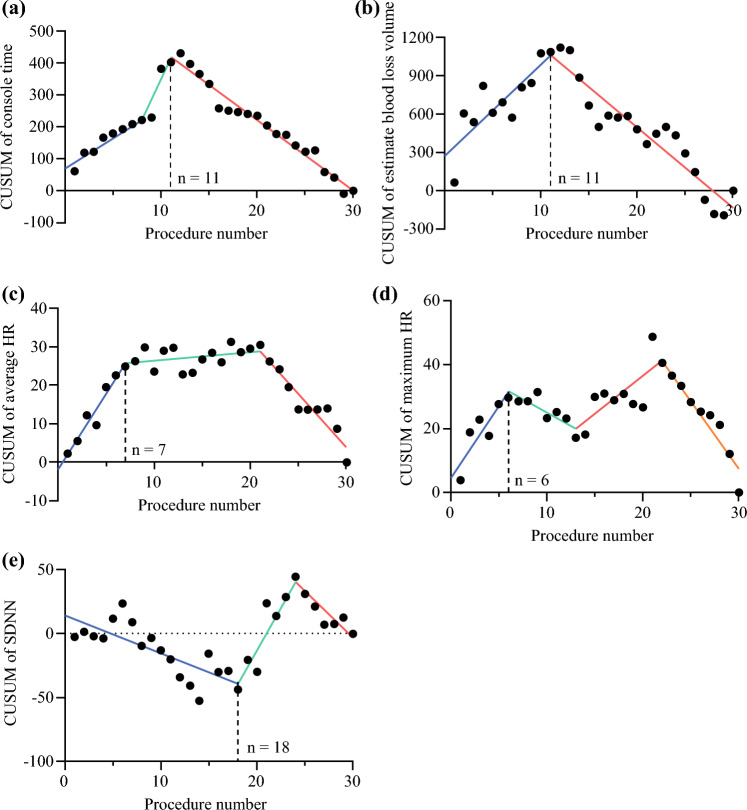
Joinpoint linear regression analysis of cumulative sum values for console time (**a**), estimated blood loss volume (**b**), and average (**c**), and maximum heart rates during surgery (**d**) of surgeon A; the inflection point of each learning curve was estimated as follows: *n* = 11, *n* = 11, *n* = 7, and *n* = 6 for console time, estimated blood loss volume, average and maximum heart rates during surgery, respectively; *CUSUM* cumulative sum controls charts, *HR* heart rate, *SDDNN* standard deviation of NN intervals

To evaluate the minute changes in surgical parameters and HRVs in each case, a scatterplot of such parameters for surgeon A was drawn. As a result, robotic console time (Fig. 3a) and estimated blood loss volume (Fig. 3b), average HR (Fig. 3c), and maximum HR (Fig. 3d) showed a significant reverse correlation with the accumulation of cases; however, only SDNN for 15 min (SDNN 15) remained unchanged (Fig. 3e). On the basis of these trends, using the CUSUM of each parameter, a learning curve analysis was performed. As a result, a transitional change past the surgeon’s learning phase was achieved by 11 cases when considering robotic console time (Fig. 4a) and estimated blood loss volume (Fig. 4b) and by 7 and 6 cases when considering average HR (Fig. 4c) and maximum HR (Fig. 4d), respectively. However, for the SDNN, the inflection point was not identified in this analysis (Fig. 4e).

### Analysis of the Impact of Factors Related to Patients on Surgeons

**Fig. 5:**
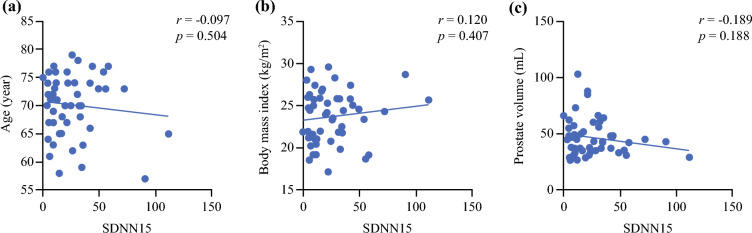
Pearson’s correlation coefficients (*r*) and *p* values between the standard deviation of NN intervals (SDNN) for 15 min, and age of patients (**a**), body mass index of patients (**b**), and prostate volume of patients (**c**)

For the analysis of risk factors concerning patients that could influence a surgeon’s HRV, those that could reduce SDNN 15 to less than the median level were analyzed. As a result, no significant tendency was found between age, BMI, and prostate volume to SDNN 15 (Fig. 5a–c). In addition, univariate logistic regression analysis revealed that these were not risk factors for the reduction of SDNN 15 (Table 2). With regard to the possibility of collider bias, a patient’s background influences the correlation between blood loss, heart rate, and number of surgeries. Therefore, a comparison of baseline characteristics of patients operated by surgeon A was made. As a result, no trends were observed in age, BMI, and prostate volume for every ten cases.

**Table 2:** Univariate logistic regression analyses of risk factors for poorer SDNN15

Parameters	OR (95% CI)	*p*-Value
Age of patients, ≥ 71 versus < 71 y	0.73 (0.24–2.21)	0.57
BMI of patients, ≥ 24.2 versus < 24.2 kg/m^2^	0.85 (0.28–2.58)	0.78
Prostate volume of patients, ≥ 44 versus < 44 ml	2.25 (0.73–6.98)	0.16
Surgeon, surgeon A versus surgeon B	3.78 (1.17–12.20)	< 0.05

*BMI* body mass index, *CI* confidence interval, *OR* odds ratio, *SDNN15* standard deviation of the NN intervals during 15 min

## Discussion

Electrocardiogram data on HR and HRV are important biomarkers of emotion. Stress in an individual induces changes in these and other physiological signals controlled by the autonomic nervous system. As prospective surrogates for ECG measurements, wearable devices equipped with PPG sensors allow the ongoing monitoring of HRV, revealing real-time stress levels and a person’s fatigue or mental health.^22,23^ Measured levels of HRV decrease when stress increases.^24^ However, the relationship between the measured HRV and mental stress in surgeons has rarely been studied.

To eliminate the influence of patient factors in advanced cases, including extensive resection or lymph node dissection, we assessed surgical outcomes of RARP in consecutive cases with a localized cT2 prostate cancer and the HRV obtained using a wearable device in both novice and expert surgeons. Above all, we examined using wearable devices to estimate the mental health of surgeons. Previously, only one report described the evaluation of surgeons using wearable devices in robotic surgery. Mazsella et al. analyzed 72 lung lobectomies undertaken by a surgeon using a Healer R2 (MDD Class IIa Medical Device, L.I.F.E. Italia S.r.l., Milan, Italy) as a wearable device, and demonstrated that competency and consistency were achieved after about 20–30 procedures.^25^ This device recorded several physiological parameters and signals, including cardiovascular and respiratory activities, and the body position of study participants. However, the sensor equipment used was relatively large-scale and heavily loaded, and its versatility remains unknown. By contrast, the Fitbit in our analysis is a widely used, wrist-worn, and commercially based device that measures movement using optical PPG and a 3D accelerometer. In addition, in terms of the assessment of mental health status, it is a device from which a wealth of data has been accumulated. For example, Nakagome et al. evaluated the HRV in 110 patients with mental illnesses using a Fitbit. They demonstrated that the HRV, including SDNN in resting time, could be a predictive factor of a patient’s mental health status.^18^

In our study, both the median maximum and average HRs, reflecting the mental stress of surgeon A, were significantly lower by 21–30 cases than by 1–10 cases. However, for surgeon B, a trend in the HRV for every ten cases was not observed. The HRVs measured by the Fitbit might be feasible for evaluation at a relatively early stage for surgeons who just started completing RARPs. Such HRVs may be considered useful as an indicator of the proficiency of a surgeon who just started performing RARPs, similar to robotic console time and estimated blood loss volume. Interestingly, among HRVs, the SDNN tended to be appropriate as an overall evaluation of the mental status between the two surgeons and to be inappropriate for the evaluation of minute changes for each detailed case during a procedure. The validation of these results is an area for future work. However, HRV data reflecting mental stress that are obtained by a Fitbit might be used to evaluate, in real time, each novice surgeon during RARP.

A learning curve is related to the period during which surgical performance improves and manifests as an increase in knowledge and skills to perform specific tasks or procedures. Generally speaking, this can be quantified by the minimum number of cases needed for each surgeon to undertake a proficient intervention.^26^ In most cases, such surgical proficiency is evaluated on the basis of surgical outcomes, including robotic console time, estimated blood loss, and perioperative or postoperative complication rates. In particular, the learning curve for surgical time, including robotic console time, in previous studies was found to cover from less than 30 cases to more than 300 cases, with differences depending on the report. This might be because the surgical difficulties varied in each case; other factors that could affect this include the communication skills of the instructing doctor or a surgical nurse’s experience. Therefore, it may be insufficient to determine the learning curve point using only a simple inflection point in a consecutive procedure. Additionally, how a learning curve was defined in RARP was not determined. We previously reported that anastomosis time and the complete resection of pT2c cases in RARP may become surgical outcomes that are able to determine the learning curve using a CUSUM method for the assessment.^20^ In our investigation, we first evaluated the learning curve of HRV obtained by a Fitbit, as well as robotic console time and estimated blood loss volume, in surgeons who initially performed RARP using a CUSUM method. We identified that the inflection points of transition beyond the surgeon’s learning phase occurred by case 11, when considering robotic console time and estimated blood loss volume, and by cases 7 and 6, when considering average HR and maximum HR, respectively, as learning curves. These results may allow us to interpret that average and maximum HRs might be perioperative parameters useful as outcomes for the evaluation of surgical proficiency similar to robotic console time and estimated blood loss volume. As one viewpoint, younger surgeons may perform RARP on patients with a higher BMI and patients who had previous abdominal surgery, which may have an influence on operative difficulty and increased blood loss, whereas the more experienced surgeon may have considered such patients inoperable and excluded them by selecting a “better” patient. Therefore, patient-related factors that could influence a surgeon’s HRVs so as to achieve less than the median level of SDNN15, were analyzed. As a result, no tendency for patient selection was found by the two surgeons (Fig. 5a–c and Table 2). However, further accumulation of data from multiple surgeons is needed. Heart rate variation, including maximum HRs and SDNN estimated by a Fitbit, might be useful outcomes for appropriate intervention such as an educational program in RARP. These findings may provide an insight into the fatigue levels and psychophysiological stress of young surgeons with a low amount of experience under real-world conditions. In addition, they may verify the acceptability, feasibility, and research potential of the Fitbit method used. Furthermore, the surgeons subjectively assessed their own stress and cognitive workloads, which were shown to correlate. Currently, data are being collected from a large number of surgeons participating across multiple institutions that may highlight concise trends in individual surgeons’ parameters and performance.

There are several limitations in this study. First, this was a pilot study of only two surgeons; the accumulation of data from multiple surgeons might be lead to a different conclusion. Second, patients’ differing backgrounds mean that this study has a potential confounding bias. Third, the CUSUM method is based on subjective judgements of visual appearances, but these grew difficult to make when cases were few. Fourth, we analyzed the absence of collider bias between the number of cases and surgeons’ outcomes; no trends were observed in age, BMI, or prostate volume for every ten cases. However, it is difficult to prove that there were no such biases at all. Further investigations at multiple institutions are warranted to support our findings.

## Conclusions

By using a convenient wearable device, mental stress, represented by maximum and average HRs, may be easily estimated and visualized in real time as surgical outcomes in RARP. These can be used to reduce learning curves for surgeons, including those for robotic console times and blood loss.

## References

[1] Lammers RL, Davenport M, Korley F, et al. Teaching and assessing procedural skills using simulation: metrics and methodology. *Acad Emerg Med*. 2008;15(11):1079–87. 18828833 10.1111/j.1553-2712.2008.00233.x

[2] Grantcharov TP, Reznick RK. Teaching procedural skills. *BMJ*. 2008;336(7653):1129–31. 18483056 10.1136/bmj.39517.686956.47 PMC2386620

[3] Arora S, Sevdalis N, Suliman I, Athanasiou T, Kneebone R, Darzi A. What makes a competent surgeon?: experts’ and trainees’ perceptions of the roles of a surgeon. *Am J Surg*. 2009;198(5):726–32. 19887202 10.1016/j.amjsurg.2009.01.015

[4] Weber J, Catchpole K, Becker AJ, Schlenker B, Weigl M. Effects of flow disruptions on mental workload and surgical performance in robotic-assisted surgery. *World J Surg*. 2018;42(11):3599–607. 29845381 10.1007/s00268-018-4689-4

[5] Menke V, Hansen O, Schmidt J, et al. The stress for surgeons: exploring stress entities with the robotic senhance surgical system. *J Robot Surg*. 2024;18(1):94. 38413542 10.1007/s11701-024-01853-6

[6] McAbee JH, Ragel BT, McCartney S, et al. Factors associated with career satisfaction and burnout among US neurosurgeons: results of a nationwide survey. *J Neurosurg*. 2015;123(1):161–273. 25679276 10.3171/2014.12.JNS141348

[7] Wetzel CM, Kneebone RL, Woloshynowych M, et al. The effects of stress on surgical performance. *Am J Surg*. 2006;191(1):5–10. 16399098 10.1016/j.amjsurg.2005.08.034

[8] Arora S, Aggarwal R, Moran A, et al. Mental practice: effective stress management training for novice surgeons. *J Am Coll Surg*. 2011;212(2):225–33. 21276534 10.1016/j.jamcollsurg.2010.09.025

[9] Prendergast C, Ketteler E, Evans G. Burnout in the plastic surgeon: implications and interventions. *Aesthet Surg J*. 2017;37(3):363–8. 28207037 10.1093/asj/sjw158

[10] Dalsgaard T, Jensen MD, Hartwell D, Mosgaard BJ, Jorgensen A, Jensen BR. Robotic surgery is less physically demanding than laparoscopic surgery: paired cross sectional study. *Ann Surg*. 2020;271(1):106–13. 29923873 10.1097/SLA.0000000000002845

[11] Park LS, Pan F, Steffens D, Young J, Hong J. Are surgeons working smarter or harder? a systematic review comparing the physical and mental demands of robotic and laparoscopic or open surgery. *World J Surg*. 2021;45(7):2066–80. 33772324 10.1007/s00268-021-06055-x

[12] Hartman SJ, Nelson SH, Myers E, et al. Randomized controlled trial of increasing physical activity on objectively measured and self-reported cognitive functioning among breast cancer survivors: the memory & motion study. *Cancer*. 2018;124(1):192–202. 28926676 10.1002/cncr.30987 PMC5735009

[13] Nogic J, Thein PM, Cameron J, Mirzaee S, Ihdayhid A, Nasis A. The utility of personal activity trackers (Fitbit Charge 2) on exercise capacity in patients post acute coronary syndrome [UP-STEP ACS Trial]: a randomised controlled trial protocol. *BMC Cardiovasc Disord*. 2017;17(1):303. 29284402 10.1186/s12872-017-0726-8 PMC5747185

[14] Rajab E, Wasif P, Doherty S, et al. Physical activity and sedentary behaviour of Bahraini people with type 2 diabetes: a cross-sectional study. *Digit Health*. 2024;10:20552076241251996. 38766358 10.1177/20552076241251997 PMC11102684

[15] Sugiyama Y, Naiki T, Tasaki Y, et al. Effectiveness of continuous monitoring by activity tracker of patients undergoing chemotherapy for urothelial carcinoma. *Cancer Treat Res Commun*. 2020;25:100245. 33291048 10.1016/j.ctarc.2020.100245

[16] Che Bakri NA, Kwasnicki RM, Giannas E, et al. The use of wearable activity monitors to measure upper limb physical activity after axillary lymph node dissection and sentinel lymph node biopsy. *Ann Surg Oncol*. 2023;30(12):7036–45. 10.1245/s10434-023-13966-7. 37507555 10.1245/s10434-023-13966-7 PMC10562272

[17] Mihaljevic AL. Group CH-NSS. Postoperative complications and mobilization following major abdominal surgery with versus without fitness tracker-based feedback (expelliarmus): a student-led multicenter randomized controlled clinical trial of the chir-net sigma study group. *Ann Surg*. 2024;280(2):202–11. 38984800 10.1097/SLA.0000000000006232 PMC11224573

[18] Nakagome K, Makinodan M, Uratani M, et al. Feasibility of a wrist-worn wearable device for estimating mental health status in patients with mental illness. *Front Psychiatry*. 2023;14:1189765. 37547203 10.3389/fpsyt.2023.1189765 PMC10399687

[19] Lovegrove C, Novara G, Mottrie A, et al. Structured and modular training pathway for robot-assisted radical prostatectomy (RARP): validation of the RARP assessment score and learning curve assessment. *Eur Urol*. 2016;69(3):526–35. 26585582 10.1016/j.eururo.2015.10.048

[20] Nagai T, Etani T, Shimizu N, et al. Learning curve of multiple surgeons for robot-assisted radical prostatectomy using the cumulative sum method: a retrospective single-institution study. *J Robot Surg*. 2024;18(1):389. 39485578 10.1007/s11701-024-02122-2

[21] Kanda Y. Investigation of the freely available easy-to-use software ‘EZR’ for medical statistics. *Bone Marrow Transplant*. 2013;48(3):452–8. 23208313 10.1038/bmt.2012.244 PMC3590441

[22] Escorihuela RM, Capdevila L, Castro JR, et al. Reduced heart rate variability predicts fatigue severity in individuals with chronic fatigue syndrome/myalgic encephalomyelitis. *J Transl Med*. 2020;18(1):4. 31906988 10.1186/s12967-019-02184-z PMC6943898

[23] Fournie C, Chouchou F, Dalleau G, Caderby T, Cabrera Q, Verkindt C. Heart rate variability biofeedback in chronic disease management: a systematic review. *Complement Ther Med*. 2021;60:102750. 34118390 10.1016/j.ctim.2021.102750

[24] Hernando D, Roca S, Sancho J, Alesanco A, Bailon R. Validation of the Apple watch for heart rate variability measurements during relax and mental stress in healthy subjects. *Sensors (Basel)*. 2018;18(8):2619. 30103376 10.3390/s18082619 PMC6111985

[25] Mazzella A, Mohamed S, Maisonneuve P, et al. Learning curve of robotic lobectomy for the treatment of lung cancer: how does it impact on the autonomic nervous system of the surgeon? *J Pers Med*. 2023;13(2):193. 36836426 10.3390/jpm13020193 PMC9961561

[26] Grivas N, Zachos I, Georgiadis G, Karavitakis M, Tzortzis V, Mamoulakis C. Learning curves in laparoscopic and robot-assisted prostate surgery: a systematic search and review. *World J Urol*. 2022;40(4):929–49. 34480591 10.1007/s00345-021-03815-1

